# HIF stabilizers in the management of renal anemia: from bench to bedside to pediatrics

**DOI:** 10.1007/s00467-017-3849-3

**Published:** 2018-03-22

**Authors:** Dalvir Kular, Iain C. Macdougall

**Affiliations:** 1 Department of Medicine, Queen’s Hospital, Romford, UK; 2 Department of Renal Medicine, King’s College Hospital, London, UK

**Keywords:** Anemia, Chronic kidney disease, HIF stabilizer, HIF prolyl-hydroxylase inhibitor

## Abstract

Anemia is a common complication of chronic kidney disease (CKD) in adult and pediatric patients. It has traditionally been treated with erythropoietin therapy and iron supplementation, with great success. With the discovery of the major transcription factor hypoxia inducible factor (HIF) for the erythropoietin gene in 1992, molecules were created that inhibit the HIF prolyl-hydroxylase enzyme. This new class of drug—called HIF stabilizers, or HIF prolyl-hydroxylase inhibitors—prevents the proteasomal degradation of HIF-α, thereby inducing upregulation of the erythropoietin gene. This new strategy for treating CKD anemia is already in phase III clinical trials in adults, and the potential advantages of this therapy are that it is orally active (thereby avoiding injections), and patients are exposed to lower circulating levels of erythropoietin. The long-term safety of this strategy, however, requires elucidation in these trials, particularly since there are many other hypoxia-sensitive genes, notably, angiogenic factors such as vascular endothelial growth factors (VEGF), as well as glycolytic enzymes. As with all new therapies, it is only once a positive benefit: risk profile has been ascertained in adults that the treatment will translate across into pediatrics. Specific issues in the pediatric CKD population are discussed in this review.

## Introduction

Anemia is a common complication of chronic kidney disease (CKD) in adult and pediatric patients; the prevalence in the latter group, as estimated from the North American Pediatric Renal Trials and Collaborative Studies (NAPRTCS), was 73% at CKD stage III, 87% at stage IV, and >93% at stage V [1]. The presence of anemia in pediatric patients with CKD is associated with decreased quality of life (QoL), reduced neurocognitive ability, left ventricular hypertrophy, increased risk of hospitalizations, an independent predictor of mortality [2–7].

Current management of anemia in CKD primarily consists of an injectable erythropoiesis-stimulating agent (ESA), administered intravenously (IV) or subcutaneously (SC), and adjuvant iron therapy orally or IV. We use the term ESA to encompass short-acting recombinant human erythropoietin (rhEPO; epoetin), medium-acting darbepoetin alfa, and long-acting epoetin beta pegol. Suboptimal response to treatment makes pediatric CKD patients vulnerable to requiring blood transfusions, which may adversely affect future transplantation through allosensitization, increase waiting time to transplant, and increase the risk of graft failure after transplant. Pediatric CKD patients are reported to be at higher risk of allosensitization than adults, and that risk increases because they are likely to have subsequent transplants in their lifetime [8, 9].

Despite the availability of ESAs, anemia in pediatric patients remains highly prevalent. Borzych-Duzalka et al., in a pediatric observational study of 1394 peritoneal dialysis (PD) patients, found that 92% were prescribed ESAs, but that 25% of patients had still had hemoglobin (Hb) levels below target (<10 or <9.5 g/dl in children older or younger than 2 years, respectively) [10]. Although ESAs are effective in most patients, a large proportion remain hyporesponsive, which in most centers leads to the escalation of ESA dose [11]. Predictors of hyporesponsiveness include secondary hyperparathyroidism, malnutrition–inflammation complex, and iron deficiency [12]. Furthermore, pediatric studies have shown that a higher ESA dose is independently associated with an increased risk of mortality in patients on dialysis [10, 13]. In addition, a meta-regression analysis of 31 randomized controlled trials of ESA use in adults found that a higher ESA dose was associated with an increased risk of all-cause mortality, hypertension, stroke, and thrombotic events independent of Hb level [14]. Apart from ESA safety concerns, recombinant ESAs are costly, and although concomitant iron supplementation may be cost-effective in reducing ESA doses, exogenous iron supplementation is not without its own problems and has been associated with an increased risk of hypersensitivity reactions, infections, and cardiovascular events [15–18].

The normal Hb range in the pediatric population varies by age, gender, and race. Furthermore, the Kidney Disease: Improving Global Outcomes (KDIGO) 2012 guidelines suggest starting an ESA on an individual basis, taking into consideration the impact on school attendance and performance, neurocognitive development, and exercise capacity [19, 20]. The current suggested target Hb range for pediatric CKD stage 5 on an ESA by KDIGO is 11–12 g/dl. The UK National Institute for Health and Care Excellence (NICE) differs slightly, recommending a target Hb for a patient on an ESA of 10–12 g/dl (age > 2) and 9.5–11.5 g/dl (age < 2), respectively [21]. Although there are observational studies that suggest avoiding an Hb <11, the upper limit of the Hb target by ESAs is debated and is informed by adult trials [22, 23].

Concerns about ESA therapy, which include increased risk of stroke, venous thromboembolism, and cancer-related mortality, have led to a search for alternative treatment options. With the unfortunate failure and voluntary recall of peginesatide, a peptide-based ESA, the next treatment on the horizon is a class of drugs known as the hypoxia inducible factor (HIF) stabilizers [24]. HIF is the major transcription factor for the* EPO* gene and represent a novel therapeutic approach to the treatment of anemia in CKD, which mimics the natural hypoxic response, by inhibiting prolyl-hydroxylase (PHD) enzymes. The consequent normoxic stabilization of HIF alpha leads to downstream pleiotropic effects, which in the pathological context of CKD promise to enhance erythropoiesis via an increase in endogenous EPO production and improved iron utilization.

In this review, we briefly discuss the pathophysiology of renal anemia then focus on the HIF pathway and its potential mechanistic role in enhancing erythropoiesis. We also discuss HIF stabilizers in development and the clinical implications of these drugs for pediatric CKD patients.

## Pathophysiology of renal anemia

The pathogenesis of renal anemia is multifactorial, although the predominant cause is a relative deficiency of EPO. Most EPO is produced by the kidneys within peritubular interstitial fibroblast-like cells and to a lesser extent by the liver and other extrarenal sites [25]. The production of EPO responds to tissue hypoxia, which stimulates EPO to maintain oxygen homeostasis through its ability to repress apoptosis, stimulate differentiation and proliferation of erythroid progenitors, and boost erythropoiesis and oxygen carrying capacity. Furthermore, in one model of CKD, it was seen that renal EPO-producing cells (REPs) contribute to the final common pathway of fibrosis through transforming into collagen-producing myofibroblasts, thereby losing their EPO-producing capacity [26]. In the later stages of CKD, patients have inappropriately low EPO levels for their degree of anemia, which cannot be overcome by extrarenal EPO synthesis [27].

The second major cause of renal anemia is iron deficiency, limiting erythropoiesis at the iron-dependent stage of Hb synthesis. Iron deficiency may be secondary to depleted iron stores (absolute iron deficiency) or impaired release of iron from body iron stores for use in erythropoiesis (functional iron deficiency). A combination of both absolute and functional iron deficiency may also be present. Most iron in the body is contained within Hb in circulating erythrocytes, and the iron requirements of erythropoiesis are largely supplied by the recycling of senescent erythrocytes from macrophages in the reticuloendothelial system and bone marrow. Dietary iron intake compensates for gastrointestinal losses of iron caused by mucosal sloughing of the order of 1–2 mg per day. Iron homeostasis is regulated by hepcidin, a peptide hormone produced by the liver, and hepcidin activity is upregulated in patients with chronic inflammation, such as occurs in CKD. This exacerbates the anemia by limiting iron availability to the bone marrow [28].

## HIF pathway

**Fig. 1:**
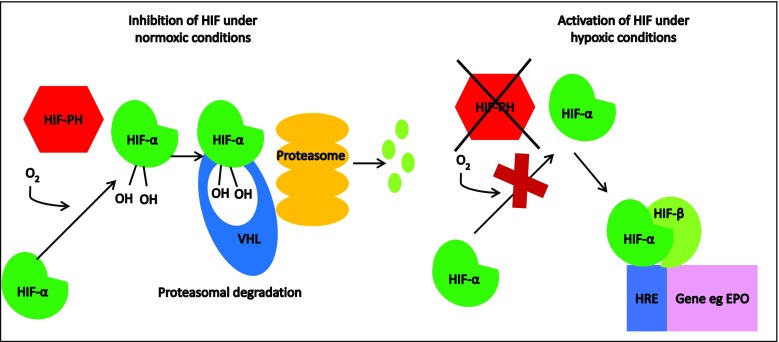
Regulation of hypoxia inducible factor (HIF) activity.* HIF-PH* hypoxia inducible factor prolyl-hydroxylase,* HIF-α* hypoxia inducible factor alpha,* HIF-β* hypoxia inducible factor beta,* HRE* hypoxia response element,* O*_*2*_ oxygen,* OH* hydroxyl group,* VHL* von Hippel-Lindau,* EPO* erythropoietin gene

The hypoxia-inducible signaling factor pathway facilitates physiological adaptation to hypoxia at a cellular level by altering gene expression. HIFs are heterodimeric transcription factors formed by the binding of α (three isoforms HIF-1α, -2α and -3α) and β subunits. The β subunit is constitutively expressed, whereas the α subunit is regulated post-translationally in an oxygen-dependent manner through the action of PHD (1, 2, and 3) enzymes. PHDs are Fe(II)- and 2-oxoglutarate-dependent dioxygenases that, under normoxic conditions, use 2-oxoglutarate (OG) as a substrate for hydroxylation of specific proline residues within HIF-α subunits. Hydroxylated HIF-α binds to tumor suppressor protein von Hippel-Lindau (p-VHL), which targets it for polyubiquitination and proteasomal degradation [29]. In contrast, under hypoxic conditions or through the pharmacological action of an HIF stabilizer (OG competitor), PHDs are inhibited and HIF-α is no longer degraded, allowing it to dimerize with the β subunit and bind to the hypoxia response elements (HREs) in >100 genes, including EPO and genes involved in iron homeostasis (Fig. 1) [30, 31].

### HIF and EPO

In vivo knockout studies have identified HIF-2α as the primary regulator of hypoxic EPO induction in both the kidney and liver. However, the role of oxygen sensor PHDs differs between both organs in EPO production, as renal interstitial cells predominantly use PHD2 and hepatocytes use all three PHD isoforms equally for degrading HIF-2α [32]. In the kidney, peritubular interstitial cells respond to a change in tissue oxygenation by turning either on or off, corresponding to their ability to produce EPO (on-REPs and off-REPs, respectively). During normoxia, there may be a greater proportion of off-REPs: however, depending on the degree of hypoxia, there is an increase in EPO production due to a greater proportion of on-REPs rather than a change in EPO expression level in each REP [26, 33, 34].

### HIF and iron homeostasis

The HIF pathway also regulates iron homeostasis to meet the iron demands of erythropoiesis via direct and indirect mechanisms. Directly, the activation of HIF-2 in the small intestine binds to the HREs of apical iron absorption genes, duodenal cytochrome b (*DCYTB*), and divalent metal transporter-1 (*DMT1*). Subsequent translation of *DCYTB* and *DMT1* allows dietary iron (Fe^3+^) to be reduced to ferrous iron (Fe^2+^) and imported into the enterocyte. This critical process is impaired in intestinal HIF-2α knockout mice during acute anemia, resulting in inadequate erythropoiesis compared with wild-type mice [35]. In addition, HIF-2 acutely upregulates ferroportin during iron deficiency in the small intestine, thereby increasing the capacity of basal iron export [36]. In contrast, HIF-1 has been shown in vitro to directly upregulate transferrin, ceruloplasmin, and transferrin receptor genes, which would function to increase iron transport and utilization [37–39].

Indirectly, the induction of EPO by HIF-2α leads to erythropoiesis-mediated suppression of hepcidin levels [40]. During erythropoiesis, erythroblasts produce erythroferrone (ERFE), a hormone that acts directly on the liver to downregulate *HAMP* transcription, inhibiting hepcidin production, and thereby increasing iron availability for efficient erythrocyte synthesis [41].

## HIF stabilizers in the pipeline

The first HIF stabilizer to reach a phase I trial was Fibrogen’s FG-2216. In a proof-of-concept study, orally bioavailable FG-2216 was shown to stimulate endogenous EPO production when given to 12 hemodialysis (HD) patients, six of whom were anephric and six healthy participants. FG-2216 increased plasma EPO levels by 30.8-fold in HD patients, 14.5-fold in anephric patients, and 12.7-fold in healthy volunteers [30]. However, during a phase II trial of FG-2216, a patient developed fulminant hepatitis and died, which suspended development of the drug due to safety concerns [42]. Despite approval of the US Food and Drug Administration (FDA) for phase II trials to continue, further development of FG-2216 by Fibrogen was voluntarily discontinued [43, 44], and the company focused on another molecule under development: FG–4592, now called roxadustat. Several pharmaceutical companies are developing orally bioavailable HIF stabilizers, which have either started phase III studies or are waiting to do so (Table 1).

**Table 1:** Phase III trials of hypoxia inducible factor (HIF) stabilizers

Drug	Company	NCT reference	Study population	No.	Active comparator(s) drug	Outcomes*	Duration	Status	Start date; estimated completion date
Roxadustat FG-4592 ASP1517	FibroGen Astellas AstraZeneca	02021318 (Dolomites)	NDD-CKD	*n* = 570	Darbepoetin alfa	**Hb response** Use of rescue therapy BP parameters Hospitalizations Lipid/Iron/HbA1c/glucose parameters Kidney function IV Iron requirements QOL Safety profile	24 weeks- Up to 108 weeks	Recruiting	03/2014–04/2020
		02273726	HD/PD	*n* = 820	Epoetin alfa	**Mean Hb change from baseline** BP parameters Lipid parameters IV Iron requirements QOL	52 weeks	Recruiting	12/2014–03/2018
		02174731	Incident and stable HD/PD	*n* = 2100	Epoetin alfa	**MACE** Time to rescue therapy Hb parameters All-cause mortality QOL Safety profile	event-driven, anticipate 1–2 years	Recruiting	07/2014–04/2018
		01887600 (ALPS)	NDD-CKD	*n* = 597	Placebo	**Mean Hb change from baseline** **Hb response** Use of rescue therapy BP parameters Lipid parameters Hospitalizations Iron/HbA1c/glucose parameters Kidney function QOL Safety profile	52 weeks- Up to 108 weeks	Active, not recruiting	09/2013–11/2017
		02174627	NDD-CKD	*n* = 2700	Placebo	**MACE** Hb parameters Kidney function QOL Time to rescue therapy All-cause mortality Safety profile	Event-driven, anticipate 1–2 years	Active, not recruiting	06/2014–03/2018
		02278341 (Pyrenees)	HD/HDF/PD	*n* = 838	Darbepoetin alfa Epoetin alfa	**Mean Hb change from baseline** BP parameters Lipid parameters IV Iron requirements QOL	28 to 52 weeks	Active, not recruiting	11/2014–07/2018
		02052310	Incident HD/PD	*n* = 900	Epoetin alfa	**Mean Hb change from baseline** BP parameters Lipid parameters IV Iron requirements	52 weeks and maximum of up to 3 years	Recruiting	12/2013–03/2018
		01750190	NDD-CKD	*n* = 900	Placebo	**Hb response and maintenance** BP parameters Lipid parameters QOL Use of rescue therapy Safety profile	52 weeks	Recruiting	11/2012–03/2018
		02988973	NDD-CKD	*n* = 325	Darbepoetin alfa	**Mean Hb change from baseline** QOL Safety profile (including ophthalmological examination)	24- up to 52 weeks	Recruiting	01/2017–11/2018
		02780726	PD (>4 weeks)	*n* = 56	N/A	**Hb response rate** QOL Safety profile	24 weeks	Active, not recruiting	06/2016–09/2017
		02964936	NDD-CKD	*n* = 100	N/A	**Hb response rate** QOL Safety profile	24 weeks	Recruiting	01/2017–05/2018
		02952092	HD	*n* = 300	Darbepoetin alfa	**Mean Hb change from baseline** QOL Safety profile (including ophthalmological examination)	24 weeks	Recruiting	11/2016–06/2018
		01630889 (Phase II/III)	NDD-CKD and DD-CKD	*n* = 50	N/A	**Long-term efficacy and safety in maintenance of Hb**	Up to 5 years	Enrolling by invitation	05/2012–12/2018
Vadadustat AKB-6548	Akebia Therapeutics	02648347 PRO_2_TECT-CORRECTION	NDD-CKD	*n* = 1000	Darbepoetin alfa	**Hb change from baseline** **MACE** Safety profile	52 weeks–min 1 year (event driven)	Recruiting	12/2015–11/2018
		02865850 INNO_2_VATE – CORRECTION	Incident HD/PD	*n* = 400	Darbepoetin alfa	**Hb change from baseline** **MACE** Safety Profile	52 weeks–min 1 year (event driven)	Recruiting	07/2016–09/2019
		02680574 PRO_2_TECT-CONVERSION	NDD-CKD	*n* = 2100	Darbepoetin alfa	**Hb change from baseline** **MACE** Safety profile	52 weeks–min 1 year (event driven)	Recruiting	02/2016–11/2018
		02892149 INNO_2_VATE-CONVERSION	HD/PD	*n* = 2200	Darbepoetin alfa	**Hb change from baseline** **MACE** Safety profile	52 weeks–min 1 year (event driven)	Recruiting	08/2016–09/2019
Daprodustat GSK1278863	GlaxosmithKline	03029208	Incident HD/PD	*n* = 300	Darbepoetin alfa	**Mean Hb change from baseline during study period** IV Iron requirement BP parameters Time to rescue therapy QOL	52 weeks	Recruiting	5/2017–11/2019
		02791763	NDD-CKD/PD	*n* = 320	Epoetin beta pegol	**Mean Hb during study period** Hb parameters Iron parameters	52 weeks	Recruiting	6/2016–6/2018
		02969655	HD	*n* = 270	Darbepoetin alfa Placebo	**Mean Hb during study period** Hb parameters	52 weeks	Active, not recruiting	11/2016–07/2018
		02879305 (ASCEND-D)	HD/PD	*n* = 3000	Epoetin alfa Darbepoetin alfa	**Hb change from baseline** **MACE** IV Iron requirement Hospitalizations BP parameters QOL	52 weeks- up to 3.3 years (event driven)	Recruiting	6/2014–04/2020
		02876835 (ASCEND-ND)	NDD-CKD	*n* = 4500	Darbepoetin alfa	**Hb change from baseline** **MACE** Hospitalizations BP parameters QOL	52 weeks-up to 4.1 years (event driven)	Recruiting	09/2016–01/2021
Molidustat BAY85–3934	Bayer Pharmaceuticals	Phase III trials not announced							

Primary outcome(s) in bold

*CKD* chronic kidney disease,* Hb* hemoglobin,* HD* hemodialysis,* IV* intravenous,* MACE *major adverse cardiovascular event,* NDD-CKD* non-dialysis-dependent CKD stage 3 or 4 * QOL* quality of life,* PD* peritoneal dialysis

### Roxadustat (FG-4592/ASP1517)

Roxadustat, also known as FG-4592 and ASP1517, is a second-generation HIF stabilizer being developed by FibroGen, Astellas, and AstraZeneca. The orally available compound chemically resembles FG-2216 but has the addition of a phenoxy group in its quinolone core. Six phase II studies have been published: three in non-dialysis-dependent (NDD)-CKD participants and three in dialysis-dependent (DD)-CKD participants. 

In a single-blind, placebo-controlled study, 117 NDD-CKD stage 3 or 4 patients were randomized to receive four escalating doses (0.7, 1.0, 1.5, 2.0 mg/kg) of roxadustat either twice or thrice weekly over 28 days [45]. The study found that roxadustat increased Hb from baseline in a dose-dependent manner. The median time to increase Hb ≥1 g/dl was shorter in the 1.5 and 2 mg/kg thrice-weekly cohorts compared with the equivalent twice-weekly cohorts. Following oral administration of 1 mg/kg roxadustat twice weekly, endogenous EPO (eEPO) levels began to rise after 4 h, peaked at ~10 h, and returned to baseline within 24–48 h. In contrast to exogenous recombinant EPO administration, peak eEPO levels with roxadustat were within the physiological range seen in acute bleeding, and the 48-h time-averaged concentration was comparable with that of high-altitude acclimation [46, 47].

In a subsequent phase IIa open label study, various roxadustat dose regimens were evaluated for 16 and 24 weeks in NDD-CKD participants (*n* = 145). The mean baseline Hb was 9.7 g/dl. Despite only 52.4% participants being iron replete [ferritin >100 ng/ml and transferrin (TSAT) >20%] and the absence of IV iron administration, roxadustat corrected and maintained Hb levels ≥11.0 g/dl in most patients. During the first 16 weeks, reticulocyte Hb content was maintained, while hepcidin levels decreased. TSAT and ferritin levels declined during the initial weeks of treatment with roxadustat but then subsequently stabilized [48].

In an open-label, phase IIb study, ESA-naïve incident PD and HD participants (total* n* = 60) with severe anemia (mean Hb 8.3 g/dl at baseline) were randomized to receive no iron, oral iron, or IV iron while being treated with roxadustat for 12 weeks [49]. Most participants were iron deficient, with a mean TSAT of 18.8% and a mean ferritin of 159 ng/ml at baseline. Roxadustat increased Hb ≥2 g/dl within 7 weeks of treatment. In addition, the Hb response was independent of baseline Hb level, iron repletion status, inflammatory status [C-reactive protein (CRP)], and dialysis modality. A greater Hb response was seen in the iron groups compared with the arm receiving no iron. However, interestingly, and in contrast to previous randomized controlled trials of iron administered IV or orally with recombinant EPO therapy, Hb response was similar regardless of administration route. Serum hepcidin levels were measured as an exploratory endpoint; at 4 weeks they had decreased significantly, with the greatest and lowest reductions seen in the no iron and IV iron supplementation dialysis arms, respectively.

Finally, in an open-label, phase II study, HD participants (*n* = 144) with Hb levels previously maintained (mean Hb ≥11 g/dl) by epoetin alfa were randomized (3:1) to receive roxadustat or continue epoetin alfa [50]. The use of IV iron was prohibited. The study consisted of two parts: In part 1 (*n* = 41), four escalating dose cohorts (1.0, 1.50, 1.80, 2.0 mg/kg thrice weekly) were compared to epoetin alfa over a period of 6 weeks. The primary endpoint was the proportion of participants who showed no reduction of Hb level by 0.5 g/dl from baseline. In the pooled roxadustat ≥1.5 mg/kg cohort, 79% of participants achieved the primary endpoint, compared with 33% in the epoetin alfa control arm. In part 2, various titratable dose cohorts (weight and tiered based) were compared with epoetin alfa over 19 weeks. The primary endpoint was the proportion of participants able to maintain Hb levels ≥11 g/dl over the last 4 weeks of the study. The average roxadustat dose required to maintain Hb levels was ~1.7 mg/kg thrice weekly (range 0.5–3.4). Similar results were observed in another conversion study of 87 HD patients (mean Hb of 10.7 g/dl at baseline) maintained on epoetin alfa prior to study entry. Participants were randomized to receive either one of three weight-based dosing regimens—low (1.1–1.8 mg/kg), medium (1.5–2.3 mg/kg), and high (1.7–2.3 mg/kg)—of roxadustat or to continue epoetin alfa over 6 weeks. Of patients randomized with low-, medium- and high doses of roxadustat, 59.1%, 88.9%, and 100%, respectively, maintained Hb levels after 5 and 6 weeks, compared with 50% in the epoetin arm [51].

In addition to Hb response and the effect on iron indices, the effects of roxadustat on total cholesterol levels have been explored in NDD-CKD and DD-CKD [48, 50, 51]. Results from studies so far show a significant reduction in total cholesterol from baseline, with the greatest reductions seen in the highest baseline tertiles [51]. In contrast, comparator groups, placebo, and epoetin alfa showed no significant change in cholesterol from baseline [50, 51].

On the whole, roxadustat was well tolerated, and adverse and serious adverse events seen in roxadustat studies were consistent with those seen in the dialysis population and similar to comparator groups. The only serious adverse event considered as possibly related to roxadustat was an episode of acute pancreatitis; however, both the sponsor and the independent data monitoring committee considered it unrelated because of a possible alternative cause [50].

Roxadustat is the most advanced HIF stabilizer in development, with phase III trials underway. Preliminary results from two phase III trials in China have been reported by FibroGen in NDD-CKD and DD-CKD participants. In the NDD-CKD study, a double-blind, placebo-controlled 8-week portion of a 26-week study, 151 participants (mean Hb 8.9 g/dl at baseline) were randomized 2:1 to receive roxadustat (*n* = 101) or placebo (*n* = 50) [52]. At 8 weeks, roxadustat-treated participants achieved a mean Hb increase of 1.9 g/dl from baseline, whereas the placebo arm achieved a mean change in Hb of −0.4 g/dl from baseline (*p* < 0.00001). A significantly higher proportion of roxadustat patients achieved an Hb increase ≥1 g/dl from baseline after 8 weeks compared with placebo participants: 84.2% vs 0.0% (*p* < 0.00001). In the DD-CKD study, 304 participants (HD and PD) previously maintained on epoetin alfa were randomized 2:1 to either roxadustat or epoeitin alfa for 26 weeks [52]. Mean Hb levels were increased to a greater extent in patients receiving roxadustat, and the drug was noninferior to the epoetin alfa arm in maintaining Hb levels. If phase III trials of roxadustat are supportive, Fibrogen expects to submit a new drug application (NDA) to the FDA in 2018 [53].

As of this writing, there have been no reported studies of roxadustat in pediatric participants. However, a pediatric investigation plan (PIP) has been agreed to by Astellas and the European Medicines Agency (EMA), with a deadline to complete studies by 2021 (Table 2) [54].

**Table 2:** Roxadustat (FG-4592/ASP1517) pediatric investigation plan (PIP)

Roxadustat (FG-4592 /ASP1517), (EMEA-001557-PIP01-13) decision: 30 January 2015	
Subset of pediatric population concerned	6 months to 18 years
Quality related studies	1. Development of lower strength of the film-coated tablet not containing azo dyes
Non-clinical studies	2. Definitive juvenile toxicity study
Clinical studies	3. Open label, randomized, 3-way, cross-over study to evaluate the relative bioavailability, PK, food effect, and palatability of the pediatric formulation versus adult tablet. 4. Open-label, randomized, age-group-adjusted starting dose, active-controlled trial to evaluate PK/PD, safety and efficacy of roxadustat compared with recombinant human EPO or its analogs in ESA-naïve children from 6 months to <18 years with anemia due to CKD stages 3, 4, and 5. 5. Open-label, randomized, age-group-adjusted starting dose, active-controlled trial to evaluate PK/PD, safety, and efficacy of roxadustat compared with recombinant human EPO or its analogs in children from 6 months to <18 years with anemia due to CKD stages 3, 4, and 5 on stable ESA treatment.
Studies to be completed by	2021

*PK* pharmacokinetics,* PD* pharmacodynamics,* EPO* erythropoietin,* ESA* erythropoiesis-stimulating agent,* CKD* chronic kidney disease

### Vadadustat

Vadadustat, also known as AKB-6548, is being developed by Akebia Therapeutics and Otsuka Pharmaceuticals [55]. Following oral administration with vadadustat, eEPO levels returned to baseline within 24 h, similar to the expected physiological diurnal response [56].

In a phase IIa, double-blind, placebo-controlled trial, NDD-CKD (*n* = 93) participants were randomized to receive escalating doses (240, 370, 500, 630 mg) of vadadustat or placebo orally once daily for 6 weeks [56]. All participants received 50 mg orally of iron daily or were able to continue IV regimens if the dose did not exceed 250 mg. Vadadustat significantly increased Hb levels in a dose-dependent manner across all doses compared with placebo; 78% of participants in the 630-mg arm achieved an Hb response ≥1 g/dl at 6 weeks. In addition, iron indices were manipulated to promote erythropoiesis in a dose-dependent manner, with decreased hepcidin and ferritin and increased total-iron-binding capacity (TIBC).

In a subsequent phase IIb, double-blind, placebo-controlled trial, 210 NDD-CKD stage tjhree to five participants were randomized 2:1 into three cohorts to receive a titratable dose of vadadustat (initial dose 450 mg) or placebo once daily for 20 weeks [57]. The three cohorts were based on Hb level and ESA treatment status at screening: cohort 1 were ESA naïve (Hb ≤10.5 g/dl); cohort 2 were previously treated with an ESA (Hb ≤10.5 g/dl), and cohort 3 were actively treated with ESA (Hb ≥9.5 to ≤12.0 g/dl). Participants were iron replete at baseline, and approximately half received supplementation orally at baseline. Iron supplementation orally was allowed in order to maintain ferritin levels between 50 and 300 ng/ml; however, if participants were intolerant, then IV supplementation was permitted. At the end of the 20 weeks, more vadadustat-treated participants achieved the primary endpoint (mean Hb level ≥ 11.0 g/dl or an increase in Hb ≥1.2 g/dl above baseline) compared with placebo (53.9% vs 10.3%, *p* < 0.001). In a post-hoc analysis, vadadustat dose requirement for correction and maintenance of Hb was independent of ESA dose at baseline and systemic inflammation (as assessed by CRP).

Comparable results were seen in a 16-week conversion study in 94 HD participants (Hb 9–12 g/dl) maintained on ESA therapy prior to study entry. Participants were iron replete at baseline, and IV iron was permitted. Participants were randomized from an ESA to 300 mg once daily, 450 mg once daily, or 450 mg thrice weekly. Mean change in Hb level within each cohort remained stable throughout the study (e.g., baseline to week 16 ranged from −0.02 to −0.04 g/dl) [57].

In contrast to roxadustat phase II studies, vadadustat administration has not been associated with a change in total cholesterol [56, 57]. In phase II studies, it was well tolerated, and adverse events were similar to the active comparator groups and typical of the CKD population.

Vadadustat is undergoing four phase III trials. These consist of a two-study program in both NDD- and DD-CKD participants, termed PRO_2_TECT (*n* = ~3100) and INNO_2_VATE (*n* = ~2600). These are global studies with a noninferiority design and include anemia correction and conversion studies, with the active comparator being darbepoetin alfa. A PIP has been agreed to by the EMA, with a deadline to complete studies by 2024 (Table 3) [58].

**Table 3:** Vadadustat (AKB-6548) pediatric investigation plan (PIP)

Vadadustat (AKB-6548), (EMEA-001944-PIP01-16) decision: 31 January 2017	
Subset of pediatric population concerned	4 months–18 years
Quality-related studies	1. Development of an age-appropriate oral dosage form
Non-clinical studies	2. Dose range-finding study in juvenile dogs 3. Definitive toxicity study in juvenile dogs
Clinical studies	4. Open-label, single-arm, externally controlled trial to evaluate the activity, safety, tolerability, PK, and PD of vadadustat orally to correct anemia in children from 4 months to <18 years of age with anemia secondary to chronic kidney disease (CKD). 5. Open-label, single-arm, externally controlled trial to evaluate activity, safety, tolerability, PK, and PD of vadadustat orally for maintenance treatment of anemia in children from 4 months to <18 years of age with anemia secondary to CKD receiving ESA treatment 6. Medical record review study to assess activity and safety of ESA treatment to maintain Hb levels in children from 4 months to <18 years of age with anemia secondary to CKD receiving ESA treatment.
Extrapolation, modeling, and simulation studies	7. Modeling and simulation study to develop PK/PD simulations to predict exposure and selection of doses for use in children from 4 months to <18 years of age with anemia secondary to CKD naïve to ESA treatment or receiving ESA treatment.
Studies to be completed by	2024

*PK* pharmacokinetics ,* PD* pharmacodynamics,* ESA* erythropoiesis-stimulating agent,* CKD* chronic kidney disease

### Daprodustat

Daprodustat, also known as GSK1278863, is being developed by GlaxoSmithKline. In a 28-day, double-blind, phase IIa study, NDD-CKD stage 3–5 participants (*n* = 73) were randomized to receive fixed daprodustat doses 0.5 mg, 2 mg, and 5 mg once daily or placebo [59]. Mean Hb at baseline ranged from 9.74 to 10.08 g/dl, and participants were iron replete. Although iron orally was allowed, IV iron was not permitted. Daprodustat demonstrated a dose-dependent increase in Hb, and the 5-mg arm resulted in a mean increase of 1 g/dl at week 4. Moreover, daprodustat produced a dose-dependent decrease in hepcidin concentrations, and the 5-mg arm saw a decrease in ferritin levels with an increase in transferrin levels and TIBC. In a parallel phase IIa conversion study, 83 HD participants maintained on stable doses of rhEPO were randomized to receive the same doses of daprodustat as the prior study or to continue rhEPO [59]. Only the 5-mg arm of daprodustat could maintain Hb levels similarly to rhEPO, whereas the lower-dose arms saw a reduction of Hb levels at 4 weeks. In addition, hepcidin levels increased in the 0.5- and 2-mg arms, stayed the same in the 5-mg arm, and decreased in the rhEPO arm at 4 weeks. Although there was a small trend of increasing TIBC and decreasing ferritin, other iron parameters were variable and did not show a clear trend.

In a 24-week, phase IIb study, daprodustat was studied in two parts in HD participants (*n* = 216) previously maintained (mean Hb 10.4 g/dl) on stable doses of rhEPO [59]. In part 1, participants were randomized to receive fixed escalating doses of daprodustat at 4, 6, 8, 10, or 12 mg or placebo once daily for 4 weeks. In part 2, after 4 weeks, doses could be titrated, and the placebo group was switched back to rhEPO, with both groups targeting an Hb of 10–11.5 g/dl for the last 20 weeks of the study. At 4 weeks, daprodustat produced dose-dependent mean changes in Hb (g/dl) from baseline (placebo −0.72; 4 mg −0.29; 6 mg 0.18; 8 mg 0.40; 10 mg 0.69; 12 mg 0.69). In part 2, at 24 weeks, mean change in Hb in the rhEPO arm and combined daprodustat group were, −0.11 vs 0.03 g/dl, respectively. In the combined daprodustat group, mean hepcidin, ferritin, and TSAT decreased, while TIBC increased compared with baseline (unpublished results) [60].

Similar to studies in roxadustat, Holdstock et al. reported modest decreases in total cholesterol with daprodustat in NDD- and DD-CKD participants. In the studies overall, daprodustat was well tolerated and safe. Phase III studies are recruiting and will include two large studies in NDD- and DD-CKD participants, termed ASCEND-ND (*n* = ~4500) and ASCEND-D (*n* = 3000) [61]. A PIP has been agreed to by EMA, with a deadline to complete studies by 2027 (Table 4) [62].

**Table 4:** Daprodustat (GSK1278863) pediatric investigation plan (PIP)

Daprodustat (GSK1278863), (EMEA-001452-PIP01-13) decision: 6 August 2014	
Subset of pediatric population concerned	1–18 years
Quality-related studies	1. Development of an oral solution or oral suspension
Non-clinical studies	2. Dose range-finding study in juvenile rats 3. Definitive toxicity study in juvenile rats
Clinical studies	4. Relative bioavailability study of oral solution or oral suspension in adults 5. Open-label, single-arm, sequential cohort trial to evaluate pharmacokinetics and safety of GSK1278863 in children from 1 to <18 years undergoing dialysis 6. Open-label, single-arm, sequential cohort trial to evaluate pharmacokinetics and safety of GSK1278863 in children from 1 to <18 years who are not undergoing dialysis 7. Open-label, randomized, dose-titration, active controlled trial to evaluate efficacy and safety of GSK1278863 in children from 1 to <18 years with anemia undergoing dialysis 8. Open-label, randomized, dose-titration, active controlled trial to evaluate efficacy and safety of GSK1278863 in children from 1 to <18 years with anemia who are not undergoing dialysis
Studies to be completed by	2027

### Molidustat

Molidustat, also known as BAY 85–3934 is being developed by Bayer, who have completed five phase II trials, the results of which two NDD-CKD trials are currently known.

In a 16-week, double-blind, placebo-controlled study, ESA-naïve NDD-CKD (stages 3–5) participants (*n* = 121) were randomized equally to receive five fixed dosages of molidustat: 25 mg once daily, 50 mg once daily, 75 mg once daily, 25 mg twice daily, 50 mg twice daily, or placebo [63]. Baseline iron status was adequate (TSAT ≥20% or ferritin level ≥ 100 μg/L), and mean Hb level at baseline was 9.5 g/dl. Molidustat increased Hb levels in a dose-dependent manner by at ≥1.14 g/dl from baseline, whereas on placebo, Hb remained stable. Additionally, in a 16-week, active comparator, phase IIb study, NDD-CKD (stages 3–5) participants (*n* = 124) previously maintained on darbepoetin were randomized to titratable dosages of molidustat (starting doses 25, 50, 75 mg) or to continue darbepoetin [64]. Molidustat successfully maintained Hb levels within the target range after switching from darbepoetin treatment. Neither of the two studies commented on the effect of molidustat on iron metabolism or inflammatory markers.

In these two studies, molidustat was generally well tolerated, and the rates of adverse events were similar. Details of plans for phase III trials have not yet been made public. A PIP has been agreed to by the EMA (Table 5) [65].

**Table 5:** Molidustat (BAY 85-3934) pediatric investigation plan (PIP)

Molidustat (BAY 85–3934), (EMEA-001546-PIP01-13) decision: 27 October 2014	
Subset of pediatric population concerned	6 months–18 years
Quality-related studies	1. Development of age-appropriate oral dosage form 2. Development of age-appropriate tablet strengths
Non-clinical studies	N/A
Clinical studies	1. Relative bioavailability and food-effect study in healthy adults 2. Open-label, randomized, active-controlled, noninferiority trial to evaluate efficacy, safety, and pharmacokinetics of molidustat compared with standard of care in ESA-naïve children from 6 months to <18 years with renal anemia (correction study) 3. Open-label, randomized, active-controlled, noninferiority trial to evaluate efficacy, safety, and pharmacokinetics of molidustat compared with standard of care in children from 6 months to <18 years with renal anemia on stable ESA treatment (maintenance study) 4. Open-label, follow-up trial to evaluate safety of molidustat in children who participated in the correction and maintenance studies
Extrapolation, modeling, and simulation studies	5. Modeling study to evaluate pharmacokinetic properties of molidustat in children from 6 months to <18 years
Studies to be completed by	2024

## Discussion

Short-term phase II data show that HIF stabilizers can correct renal anemia and maintain Hb concentration with once-daily to thrice-weekly dosing in both NDD-CKD and DD-CKD patients.

There are multiple potential advantages of HIF stabilizers over current treatment. First, oral administration is attractive when considering compliance and convenience, particularly in PD and NDD-CKD patients. Moreover, the avoidance of pain and discomfort associated with injections is particularly relevant for pediatric patients in whom subcutaneous, longer-acting ESA, darbepoetin, has been associated with significantly greater pain perception compared with short-acting ESA, epoetin beta [66]. Second, HIF stabilizers are less expensive to produce than ESAs for several reasons, with the manufacturing process relying on synthetic chemistry rather than recombinant DNA technology. There is also a reduced need for sterile manufacturing conditions and no need for cold-chain transport due to stability at room temperature. In addition, the absence of a protein structure also removes concerns regarding immunogenicity, a problem seen in post-approval epidemiological studies of ESAs [67]. Third, phase II data provide evidence that HIF stabilization increases iron availability for erythropoiesis, with HIF stabilizers being effective independent of iron repletion status, inflammation status, and route of exogenous iron delivery. The clinical consequences of this may preclude the need for iron supplementation in certain patients, at least in the short term, and reduce the need for IV iron, with potential safety concerns. Finally, other possible class benefits are being explored, particularly outcomes such as lipids and blood pressure. With the former, both daprodustat and roxadustat, but not vadadustat, have been shown to reduce lipids, with no published data on molidustat. There is very limited evidence to support any role in reducing blood pressure, although this will be explored in phase III trials [68].

The pleiotropic effects of the HIF pathway, however, do raise some potential concerns regarding safety, which will be addressed in the phase III trials. To understand the possible long-term consequences of the pharmacological inhibition of PHDs, one can observe pathological problems of conditions resulting from genetic mutations, high-altitude sojourners and natives, and in vitro and in vivo studies. Individuals with Chuvash polycythemia, with homozygous *VHL* mutations, for example, develop pulmonary hypertension, as do individuals with activating mutations of *HIF2A* [69, 70]. In a small, 5-day study of healthy volunteers, daprodustat was not associated with any significant rise in pulmonary artery systolic pressure measured by echocardiography [71]. The theoretical association of HIF stabilizers and tumor growth is highly complex due to the differential role of PHD and HIF isoforms, depending on tumor type. Potential upregulation of the vascular endothelial growth factor (*VEGF*) gene as a result of HIF stabilization could adversely impact tumor growth and accelerate proliferative retinopathy in diabetics. Phase II studies in vadadustat and daprodustat have not demonstrated any significant change in VEGF, and in a VEGF-sensitive model of spontaneous breast cancer, roxadustat was not associated with tumor initiation, progression, or metastases [68, 72].

A potential concern of HIF prolyl-hydroxylase inhibition of particular relevance to the pediatric population is its effect on bone and cartilage growth, as the HIF pathway is essential for normal endochondral growth-plate development. Conditional deletion of HIF-1α in murine fetal chondrocytes leads to marked shortening of limbs and massive cell death in the inner zone of the developing growth plate. In contrast to EPO induction, HIF-1α rather than HIF-2α, is critical for growth-plate development [73]. Focusing on secondary ossification, conditional deletion of PHD2 in murine chondrocytes promotes HIF-1α signaling, accelerating chondrocyte differentiation and endochondral bone formation [74]. It would be difficult to extrapolate any of these findings to different age groups within pediatric CKD given the already prevalent growth failure and the likely dose- and duration-dependent effects of PHD inhibitors [75].

As shown in Table 1, there are robust and comprehensive phase III programs planned and underway for roxadustat, vadadustat, and daprodustat, the design of which include placebo and active treatment comparisons, in both ND-CKD and DD-CKD patient populations. In addition, sample sizes of thousands of patients will have the statistical power to evaluate hard endpoints, such as all-cause mortality and cardiovascular events, with event-driven trials lasting for several years. Post-approval pharmacovigilance initiatives will be required to address the theoretical concerns of cancer incidence and late serious adverse reactions, which may appear over time and in more diverse patient groups. The latter includes patients with chronic allograft nephropathy, who have been excluded from phase III studies and are already subject to an increased risk of malignancy as a result of long-term immunosuppression [76–78].

If phase III trials are successful in establishing the safety and efficacy of the various HIF stabilizers, roxadustat will become the first HIF stabilizer to become licensed for use in adults by 2018, closely followed by vadadustat and daprodustat. The extrapolation of adult data and usage of adult formulations toward off-label and unlicensed pediatric prescribing carries risks serious adverse events in the pediatric population. Furthermore, EU regulation has mandated pediatric studies to acquire marketing authorization for HIF stabilizers in adults, which have been proposed in pediatric investigation plans (Tables 2–5). Astellas (roxadustat), Akebia (vadadustat), GlaxoSmithKline (daprodustat), and Bayer (molidustat) have all deferred pediatric studies until phase III trials have been completed, which will allow changes to be made to the design of proposed studies, if needed. There is a variety of reasons why trials in pediatric CKD patients may differ from adults. The causes of renal disease in the pediatric setting are very different from in the adult population, being largely due to congenital anomalies of the kidney and urinary tract (CAKUT), glomerulonephritis, and hereditary nephropathies; in adults, there is a huge incidence of diabetes and hypertension causing nephropathy. In addition, atherosclerotic heart disease, acute myocardial infarction, and stroke are common in adults but are uncommon in pediatric CKD [21, 72]. Thus, using these events as part of a trial’s primary endpoint is likely to be both inappropriate and futile.

The pediatric investigation plans have dictated the development of new pediatric formulations, juvenile animal toxicity studies, pharmacodynamic and pharmacokinetic studies in children, and clinical active-comparator studies assessing efficacy and safety in pediatric CKD patients aged 4 months to 18 years. The timeline of these studies gives the pediatric nephrologist some indication into the potentially wider use of these therapies, which will offer more choice in treating anemia in CKD. A likely limitation of these studies will be their short study durations, which may be compounded by high attrition rates contributed to by a high rate of transplantation in the pediatric CKD population, which would limit the collection of long-term data on effects upon growth and development.

## References

[1] Atkinson MA, Martz K, Warady BA, Neu AM (2010). Risk for anemia in pediatric chronic kidney disease patients: a report of NAPRTCS. Pediatr Nephrol.

[2] Staples AO, Wong CS, Smith JM, Gipson DS, Filler G, Warady BA, Martz K, Greenbaum LA (2009). Anemia and risk of hospitalization in pediatric chronic kidney disease. Clin J Am Soc Nephrol.

[3] Warady BA, Ho M (2003). Morbidity and mortality in children with anemia at initiation of dialysis. Pediatr Nephrol.

[4] Gerson A, Hwang W, Fiorenza J, Barth K, Kaskel F, Weiss L, Zelikovsky N, Fivush B, Furth S (2004). Anemia and health-related quality of life in adolescents with chronic kidney disease. Am J Kidney Dis.

[5] Hooper SR, Gerson AC, Johnson RJ, Mendley SR, Shinnar S, Lande MB, Matheson MB, Gipson DS, Morgenstern B, Warady BA, Furth SL (2016). Neurocognitive, social-behavioral, and adaptive functioning in preschool children with mild to moderate kidney disease. J Dev Behav Pediatr.

[6] Mitsnefes MM, Kimball TR, Kartal J, Witt SA, Glascock BJ, Khoury PR, Daniels SR (2006). Progression of left ventricular hypertrophy in children with early chronic kidney disease: 2-year follow-up study. J Pediatr.

[7] Matteucci MC, Wuhl E, Picca S, Mastrostefano A, Rinelli G, Romano C, Rizzoni G, Mehls O, de Simone G, Schaefer F (2006). Left ventricular geometry in children with mild to moderate chronic renal insufficiency. J Am Soc Nephrol.

[8] Scornik JC, Pfaff WW, Howard RJ, Fennell RS, Ramos E, Peterson JC, Neiberger R (1994). Increased antibody responsiveness to blood transfusions in pediatric patients. Transplantation.

[9] Scornik JC, Bromberg JS, Norman DJ, Bhanderi M, Gitlin M, Petersen J (2013). An update on the impact of pre-transplant transfusions and allosensitization on time to renal transplant and on allograft survival. BMC Nephrol.

[10] Borzych-Duzalka D, Bilginer Y, Ha IS, Bak M, Rees L, Cano F, Munarriz RL, Chua A, Pesle S, Emre S, Urzykowska A, Quiroz L, Ruscasso JD, White C, Pape L, Ramela V, Printza N, Vogel A, Kuzmanovska D, Simkova E, Muller-Wiefel DE, Sander A, Warady BA, Schaefer F (2013). Management of anemia in children receiving chronic peritoneal dialysis. J Am Soc Nephrol.

[11] Bamgbola OF, Kaskel FJ, Coco M (2009). Analyses of age, gender and other risk factors of erythropoietin resistance in pediatric and adult dialysis cohorts. Pediatr Nephrol.

[12] Kalantar-Zadeh K, Lee GH, Miller JE, Streja E, Jing J, Robertson JA, Kovesdy CP (2009) Predictors of hyporesponsiveness to erythropoiesis-stimulating agents in hemodialysis patients. Am J Kidney Dis 53:823–834 10.1053/j.ajkd.2008.12.040 PMC2691452 19339087

[13] Lestz RM, Fivush BA, Atkinson MA (2014). Association of higher erythropoiesis stimulating agent dose and mortality in children on dialysis. Pediatr Nephrol.

[14] Koulouridis I, Alfayez M, Trikalinos TA, Balk EM, Jaber BL (2013). Dose of erythropoiesis-stimulating agents and adverse outcomes in CKD: a metaregression analysis. Am J Kidney Dis.

[15] Prabhu MV, Nayak A, Sridhar G, Subhramanyam SV, Nayak KS (2012). Maximizing the erythropoietin response: iron strategies. Contrib Nephrol.

[16] Coyne DW, Kapoian T, Suki W, Singh AK, Moran JE, Dahl NV, Rizkala AR (2007). Ferric gluconate is highly efficacious in anemic hemodialysis patients with high serum ferritin and low transferrin saturation: results of the dialysis Patients' response to IV iron with elevated Ferritin (DRIVE) study. J Am Soc Nephrol.

[17] Kapoian T, O'Mara NB, Singh AK, Moran J, Rizkala AR, Geronemus R, Kopelman RC, Dahl NV, Coyne DW (2008). Ferric gluconate reduces epoetin requirements in hemodialysis patients with elevated ferritin. J Am Soc Nephrol.

[18] Jelkmann W (2012). Biosimilar recombinant human erythropoietins ("epoetins") and future erythropoiesis-stimulating treatments. Expert Opin Biol Ther.

[19] Hollowell JG, van Assendelft OW, Gunter EW, Lewis BG, Najjar M, Pfeiffer C (2005). Hematological and iron-related analytes--reference data for persons aged 1 year and over: United States, 1988-94. Vital Health Stat.

[20] KDIGO (2012). Clinical practice guideline for anemia in chronic kidney disease. Kidney Int Suppl.

[21] NICE guideline (2015) Chronic kidney disease: managing anaemia. NG8. Available from www.nice.org.uk

[22] Hattori M (2017). Hemoglobin target in children with chronic kidney disease: valuable new information. Kidney Int.

[23] Rheault MN, Molony JT, Nevins T, Herzog CA, Chavers BM (2017). Hemoglobin of 12 g/dl and above is not associated with increased cardiovascular morbidity in children on hemodialysis. Kidney Int.

[24] Hermanson T, Bennett CL, Macdougall IC (2016). Peginesatide for the treatment of anemia due to chronic kidney disease - an unfulfilled promise. Expert Opin Drug Saf.

[25] Jelkmann W (2011). Regulation of erythropoietin production. J Physiol.

[26] Souma T, Yamazaki S, Moriguchi T, Suzuki N, Hirano I, Pan X, Minegishi N, Abe M, Kiyomoto H, Ito S, Yamamoto M (2013). Plasticity of renal erythropoietin-producing cells governs fibrosis. J Am Soc Nephrol.

[27] de Seigneux S, Lundby AK, Berchtold L, Berg AH, Saudan P, Lundby C (2016). Increased synthesis of liver erythropoietin with CKD. J Am Soc Nephrol.

[28] Malyszko J, Mysliwiec M (2007). Hepcidin in anemia and inflammation in chronic kidney disease. Kidney Blood Press Res.

[29] Kaelin WG, Ratcliffe PJ (2008). Oxygen sensing by metazoans: the central role of the HIF hydroxylase pathway. Mol Cell.

[30] Bernhardt WM, Wiesener MS, Scigalla P, Chou J, Schmieder RE, Gunzler V, Eckardt KU (2010). Inhibition of prolyl hydroxylases increases erythropoietin production in ESRD. J Am Soc Nephrol.

[31] Weidemann A, Johnson RS (2008). Biology of HIF-1alpha. Cell Death Differ.

[32] Suzuki N, Yamamoto M (2016). Roles of renal erythropoietin-producing (REP) cells in the maintenance of systemic oxygen homeostasis. Pflugers Arch.

[33] Koury ST, Koury MJ, Bondurant MC, Caro J, Graber SE (1989). Quantitation of erythropoietin-producing cells in kidneys of mice by in situ hybridization: correlation with hematocrit, renal erythropoietin mRNA, and serum erythropoietin concentration. Blood.

[34] Obara N, Suzuki N, Kim K, Nagasawa T, Imagawa S, Yamamoto M (2008). Repression via the GATA box is essential for tissue-specific erythropoietin gene expression. Blood.

[35] Anderson ER, Xue X, Shah YM (2011). Intestinal hypoxia-inducible factor-2alpha (HIF-2alpha) is critical for efficient erythropoiesis. J Biol Chem.

[36] Taylor M, Qu A, Anderson ER, Matsubara T, Martin A, Gonzalez FJ, Shah YM (2011). Hypoxia-inducible factor-2alpha mediates the adaptive increase of intestinal ferroportin during iron deficiency in mice. Gastroenterology.

[37] Rolfs A, Kvietikova I, Gassmann M, Wenger RH (1997). Oxygen-regulated transferrin expression is mediated by hypoxia-inducible factor-1. J Biol Chem.

[38] Tacchini L, Bianchi L, Bernelli-Zazzera A, Cairo G (1999). Transferrin receptor induction by hypoxia. HIF-1-mediated transcriptional activation and cell-specific post-transcriptional regulation. J Biol Chem.

[39] Mukhopadhyay CK, Mazumder B, Fox PL (2000). Role of hypoxia-inducible factor-1 in transcriptional activation of ceruloplasmin by iron deficiency. J Biol Chem.

[40] Liu Q, Davidoff O, Niss K, Haase VH (2012). Hypoxia-inducible factor regulates hepcidin via erythropoietin-induced erythropoiesis. J Clin Invest.

[41] Kautz L, Jung G, Valore EV, Rivella S, Nemeth E, Ganz T (2014). Identification of erythroferrone as an erythroid regulator of iron metabolism. Nat Genet.

[42] (2007) Adverse event of FG-2216 for the treatment of anemia. Available at: https://www.astellas.com/en/corporate/news/pdf/070507_eg.pdf. Accessed 30 June 2017

[43] Rabinowitz MH (2013). Inhibition of hypoxia-inducible factor prolyl hydroxylase domain oxygen sensors: tricking the body into mounting orchestrated survival and repair responses. J Med Chem.

[44] Astellas (2008) The FDA accepts the complete response for clinical holds of FG-2216*/FG-4592 for the treatment of anemia. Available at https://www.astellas.com/en/corporate/news/pdf/080402_eg.pdf. Accessed 1 July 2017

[45] Besarab A, Provenzano R, Hertel J, Zabaneh R, Klaus SJ, Lee T, Leong R, Hemmerich S, Yu KH, Neff TB (2015). Randomized placebo-controlled dose-ranging and pharmacodynamics study of roxadustat (FG-4592) to treat anemia in nondialysis-dependent chronic kidney disease (NDD-CKD) patients. Nephrol Dial Transplant.

[46] Ross RP, McCrea JB, Besarab A (1994). Erythropoietin response to blood loss in hemodialysis patients in blunted but preserved. ASAIO J.

[47] Milledge JS, Cotes PM (1985). Serum erythropoietin in humans at high altitude and its relation to plasma renin. J Appl Physiol.

[48] Provenzano R, Besarab A, Sun CH, Diamond SA, Durham JH, Cangiano JL, Aiello JR, Novak JE, Lee T, Leong R, Roberts BK, Saikali KG, Hemmerich S, Szczech LA, Yu KH, Neff TB (2016). Oral hypoxia-inducible factor Prolyl Hydroxylase inhibitor Roxadustat (FG-4592) for the treatment of anemia in patients with CKD. Clin J Am Soc Nephrol.

[49] Besarab A, Chernyavskaya E, Motylev I, Shutov E, Kumbar LM, Gurevich K, Chan DT, Leong R, Poole L, Zhong M, Saikali KG, Franco M, Hemmerich S, Yu KH, Neff TB (2016). Roxadustat (FG-4592): correction of anemia in incident dialysis patients. J Am Soc Nephrol.

[50] Provenzano R, Besarab A, Wright S, Dua S, Zeig S, Nguyen P, Poole L, Saikali KG, Saha G, Hemmerich S, Szczech L, Yu KH, Neff TB (2016). Roxadustat (FG-4592) versus Epoetin Alfa for anemia in patients receiving maintenance Hemodialysis: a phase 2, randomized, 6- to 19-week, open-label, active-comparator, dose-ranging, safety and exploratory efficacy study. Am J Kidney Dis.

[51] Chen N, Qian J, Chen J, Yu X, Mei C, Hao C, Jiang G, Lin H, Zhang X, Zuo L, He Q, Fu P, Li X, Ni D, Hemmerich S, Liu C, Szczech L, Besarab A, Neff TB, Peony Yu KH, Valone FH (2017). Phase 2 studies of oral hypoxia-inducible factor prolyl hydroxylase inhibitor FG-4592 for treatment of anemia in China. Nephrol Dial Transplant.

[52] FibroGen’s Roxadustat (FG-4592) meets primary endpoints in two phase 3 anemia studies in China. Available at: http://investor.fibrogen.com/phoenix.zhtml?c=253783&p=irol-newsArticle&ID=2240513. Accessed 1 July 2017

[53] Becker K, Saad M (2017). A new approach to the Management of Anemia in CKD patients: a review on Roxadustat. Adv Ther.

[54] European Medicines Agency (EMA) roxadustat PIP decision. Available at: http://www.ema.europa.eu/docs/en_GB/document_library/PIP_decision/WC500183791.pdf. Accessed 31 July 2017

[55] Akebia and Otsuka Pharmaceutical Announce Collaboration to Develop and Commercialize Vadadustat in the U.S. Available at: https://www.otsuka-us.com/discover/articles-985. Accessed 1 July 2017

[56] Martin ER, Smith MT, Maroni BJ, Zuraw QC, deGoma EM (2017). Clinical trial of Vadadustat in patients with anemia secondary to stage 3 or 4 chronic kidney disease. Am J Nephrol.

[57] Pergola PE, Spinowitz BS, Hartman CS, Maroni BJ, Haase VH (2016). Vadadustat, a novel oral HIF stabilizer, provides effective anemia treatment in nondialysis-dependent chronic kidney disease. Kidney Int.

[58] European Medicines Agency (EMA) vadadustat PIP decision. Available at: http://www.ema.europa.eu/docs/en_GB/document_library/PIP_decision/WC500223972.pdf. Accessed 31 July 2017

[59] Holdstock L, Meadowcroft AM, Maier R, Johnson BM, Jones D, Rastogi A, Zeig S, Lepore JJ, Cobitz AR (2016). Four-week studies of oral hypoxia-inducible factor-Prolyl Hydroxylase inhibitor GSK1278863 for treatment of anemia. J Am Soc Nephrol.

[60] GSK Medicine: GSK1278863. Available at: https://www.gsk-clinicalstudyregister.com/files2/gsk-113633-clinical-study-result-summary.pdf. Accessed 1 July 2017

[61] GSK starts phase III programme with daprodustat for anaemia associated with chronic kidney disease. Available at: http://us.gsk.com/en-us/media/press-releases/2016/gsk-starts-phase-iii-programme-with-daprodustat-for-anaemia-associated-with-chronic-kidney-disease/. Accessed 1 July 2017

[62] European Medicines Agency (EMA) Daprodustat PIP °ion. Available at: http://www.ema.europa.eu/docs/en_GB/document_library/PIP_decision/WC500171723.pdf. Accessed 31 July 2017

[63] Macdougall IC, Akizawa T, Berns J, Lentini S, Bernhardt T (2016). SO036 Molidustat increases hemoglobin in erythropoiesis stimulating agents (ESA)-naive anaemic patients with chronic kidney disease not on dialysis (CKD-ND). Nephrol Dial Transplant.

[64] Macdougall IC, Akizawa T, Berns J, Lentini S, Bernhardt T, Krüger T (2016). SP309 safety and efficacy of Molidustat in erythropoiesis stimulating agents (ESA) pre-treated anaemic patients with chronic kidney disease not on dialysis (CKD-ND). Nephrol Dial Transplant.

[65] European Medicines Agency (EMA) Molidustat PIP Decision. Available at: http://www.ema.europa.eu/docs/en_GB/document_library/PIP_decision/WC500178034.pdf. Accessed 31 July 2017

[66] Schmitt CP, Nau B, Brummer C, Rosenkranz J, Schaefer F (2006). Increased injection pain with darbepoetin-alpha compared to epoetin-beta in paediatric dialysis patients. Nephrol Dial Transplant.

[67] Macdougall IC, Casadevall N, Locatelli F, Combe C, London GM, Di Paolo S, Kribben A, Fliser D, Messner H, McNeil J, Stevens P, Santoro A, De Francisco AL, Percheson P, Potamianou A, Foucher A, Fife D, Merit V, Vercammen E (2015). Incidence of erythropoietin antibody-mediated pure red cell aplasia: the prospective immunogenicity surveillance registry (PRIMS). Nephrol Dial Transplant.

[68] Yousaf F, Spinowitz B (2016). Hypoxia-inducible factor stabilizers: a new avenue for reducing BP while helping hemoglobin?. Curr Hypertens Rep.

[69] Gale DP, Harten SK, Reid CD, Tuddenham EG, Maxwell PH (2008). Autosomal dominant erythrocytosis and pulmonary arterial hypertension associated with an activating HIF2 alpha mutation. Blood.

[70] Kapitsinou PP, Rajendran G, Astleford L, Michael M, Schonfeld MP, Fields T, Shay S, French JL, West J, Haase VH (2016). The endothelial Prolyl-4-Hydroxylase domain 2/hypoxia-inducible factor 2 Axis regulates pulmonary artery pressure in mice. Mol Cell Biol.

[71] Demopoulos L (2014). Lack of correlation between PK and changes in pulmonary artery systolic pressure in healthy volunteers following administration of the HIF-prolyl hydroxylase inhibitor, GSK1278863. ACCP Annual Meeting Texas.

[72] Seeley TW, Sternlicht MD, Klaus SJ, Neff TB, Liu DY (2017). Induction of erythropoiesis by hypoxia-inducible factor prolyl hydroxylase inhibitors without promotion of tumor initiation, progression, or metastasis in a VEGF-sensitive model of spontaneous breast cancer. Hypoxia (Auckl).

[73] Schipani E, Mangiavini L, Merceron C (2015). HIF-1alpha and growth plate development: what we really know. BoneKEy reports.

[74] Cheng S, Aghajanian P, Pourteymoor S, Alarcon C, Mohan S (2016). Prolyl Hydroxylase domain-containing protein 2 (Phd2) regulates Chondrocyte differentiation and secondary ossification in mice. Sci Rep.

[75] Bacchetta J, Harambat J, Cochat P, Salusky IB, Wesseling-Perry K (2012). The consequences of chronic kidney disease on bone metabolism and growth in children. Nephrol Dial Transplant.

[76] Ploos van Amstel S, Vogelzang JL, Starink MV, Jager KJ, Groothoff JW (2015). Long-term risk of cancer in survivors of pediatric ESRD. Clin J Am Soc Nephrol.

[77] Gjertson DW, Cecka JM (2001). Determinants of long-term survival of pediatric kidney grafts reported to the united network for organ sharing kidney transplant registry. Pediatr Transplant.

[78] Bartosh SM, Leverson G, Robillard D, Sollinger HW (2003). Long-term outcomes in pediatric renal transplant recipients who survive into adulthood. Transplantation.

